# Testing a syndemics perspective on the effects of multiple adversities on depression and anxiety symptoms in a representative population sample

**DOI:** 10.1007/s00127-024-02638-w

**Published:** 2024-03-14

**Authors:** Philip J. Batterham, Amy Dawel, Kristen Murray, Yiyun Shou, Amelia Gulliver, Nicolas Cherbuin, Louise M. Farrer

**Affiliations:** 1 Centre for Mental Health Research, College of Health and Medicine, The Australian National University, 63 Eggleston Road, Canberra, ACT 2601 Australia; 2 School of Medicine and Psychology, College of Health and Medicine, The Australian National University, Canberra, Australia; 3 Saw Swee Hock School of Public Health, National University of Singapore and National University Health System, Singapore, Singapore; 4 Lloyd’s Register Foundation Institute for The Public Understanding of Risk, National University of Singapore, Singapore, Singapore; 5 Centre for Research on Ageing, Health and Wellbeing, College of Health and Medicine, The Australian National University, Canberra, Australia

**Keywords:** Depression, Anxiety, Adversity, Syndemics

## Abstract

**Purpose:**

Considerable empirical evidence indicates that stressful life experiences may have a negative impact on mental health. However, it is unclear how multiple adverse experiences may intersect to influence symptoms of depression and anxiety. Using a syndemics approach to identify potential synergistic effects between major stressors, we aimed to quantify the roles of multiple recent adverse life experiences on depression and anxiety symptoms.

**Methods:**

A population-representative sample of 1090 Australian adults (53% women, M_age_ 47 years) completed a cross-sectional survey in 2022 that assessed mental health and retrospective reports of nine specific stressful life experiences in the past year.

**Results:**

The most common adverse life experiences in the past year were financial problems (64%), loneliness (63%), or a major health problem (51%). In multivariate logistic regression analyses, financial problems, personal health problems, health problems in a close contact, relationship problems and loneliness were significantly associated with both depression and anxiety symptoms (*p* < 0.05). There was just one synergistic interaction and one buffering interaction of combined adversities on anxiety, and no synergistic interactions of adverse experiences on depression. The perceived impact of combined adversities was associated with both depression (*b* = 0.59, *p* < 0.001) and anxiety (*b* = 0.48, *p* < 0.001).

**Conclusion:**

Adversity was strongly associated with depression and anxiety. Inconsistent with a syndemics framework, there were very few synergistic relationships between different types of adversities, suggesting that different adverse experiences may independently influence mental health. The findings indicate important opportunities for early intervention to prevent depression and anxiety during difficult times.

**Supplementary Information:**

The online version contains supplementary material available at 10.1007/s00127-024-02638-w.

## Introduction

Mental health problems are highly prevalent and typically have a complex etiology comprising social, psychological, cultural, and biological factors [19, 28]. There is considerable evidence that stressful life events, which are characterised by experiences in the environment that pose an objective and significant threat to an individual [5], are associated with increases in symptoms of depression and anxiety [5, 8, 12, 17, 26] and are also associated with brain differences in regions implicated in emotion regulation [4]. Studies have also highlighted a range of specific life stressors in symptoms of depression and anxiety, including unemployment [1], relationship difficulties [16, 26], bereavement [30], natural disasters [2], public health emergencies [6], financial problems [10], and loneliness [17, 26]. Furthermore, evidence for a dose–response relationship has been observed, suggesting that exposure to multiple life events has additive impacts on poorer mental health [13].

However, limited research has examined the interrelationships between stressful life events and mental health outcomes. An emerging theoretical framework called “syndemics” posits that multiple, co-occurring adverse conditions have synergistic influences on health. These conditions may relate to the experience of two or more disease states interacting synergistically in the one person, or more broadly, to interactions between the social conditions and the health of individuals and populations [21, 22]. Specifically, interactions between co-occurring social, political, economic, or environmental conditions are highly deleterious to human health, particularly in the presence of psychological or biological vulnerabilities [21]. These specific conditions may include long-term or short-term events, such as natural disasters, climate change, or health disasters (e.g., pandemics), along with broader situational factors such as financial disadvantage and relationship problems. Syndemics may also be conceptualised as the intersection between biological and social epidemics, which are both modifiable [29].

There is some evidence of syndemics relating to mental health, especially in lower-middle income countries. Depression has been proposed to have synergistic relationships with diabetes [18] and tuberculosis [25]. Depression and anxiety are also proposed to have synergistic relationships with substance use, homelessness and violence [7], intimate partner violence, and HIV/AIDS [11]. However, few studies have examined the roles of co-occurring adverse conditions on mental health, especially interactions between specific adverse environmental, social, or biological contexts using a syndemics framework. Major public health challenges such as the COVID-19 pandemic have emerged in recent years and coincided with the acceleration of climate change, which is increasing in catastrophic natural disasters, along with increasing economic inequality and international conflict [9]. Recent research has explored the synergistic relationships between the pandemic, mental health, and racism or sexual orientation (e.g., [14, 20]), but further work is needed to examine a more diverse range of environmental, social, and other conditions. The current context provides an urgency to better understand how multiple adverse experiences might influence mental health outcomes in the general population, and whether these experiences may be synergistic.

The present study aimed to test the influence of recent experiences of nine specific adverse states on symptoms of depression and anxiety in a large population sample of Australian adults. We first aimed to assess which stressful experiences had the strongest independent effect on mental health symptoms, and whether the combined impact of overlapping social and biological states further explained variance in mental health symptoms. It was hypothesised that all nine adverse experiences considered would have significant independent effects on depression and anxiety, and that the additive impact of these experiences would have a strong association with symptoms. Second, we aimed to provide a test of the syndemics framework in mental health, by identifying whether interactions between specific adverse experiences explained significant variance in depression or anxiety symptoms. Based on definitions of syndemics, it was hypothesized that the adverse experiences would consistently demonstrate significant synergistic interactions, such that experiencing a combination of any two social or biological states would be associated with significantly higher symptoms of depression and anxiety.

## Method

### Participants and procedure

A representative sample of Australian adults aged 18 years and over was recruited in May 2022 through online market research panels accessed using Qualtrics Research Services. Quota sampling was used to obtain a sample that was representative for age group, gender, and location including both State/Territory and rurality. Participants completed a 20-min online survey on the Qualtrics online survey platform. Informed consent was obtained online from all participants prior to participation in the study by selecting ‘yes’ to a question asking if they understood the information presented on the study and agreed to participate. The ethical aspects of the study were approved by The Australian National University Human Research Ethics Committee (protocol 2020/152).

### Measures

Depression symptoms were assessed using the Patient Health Questionnaire-9 (PHQ-9; [23]), which consists of nine items assessing symptoms of depression. Anxiety symptoms were measured with the Generalized Anxiety Disorder-7 (GAD-7; [24]), a seven-item scale. Both measures have exhibited strong psychometric properties, including with high criterion validity for diagnosis of major depressive disorder and generalised anxiety disorder respectively based on a cut point of scores ≥ 10 for each scale [15]. For each scale, participants were asked to evaluate the frequency of experiencing each symptom over the last two weeks, with responses ranging from not at all (0) to nearly every day (3). The PHQ-9 (Cronbach α = 0.93) and GAD-7 (α = 0.95) had high internal consistency in the current study.

Adverse life experiences in the past year were assessed with a bespoke index that was adapted from the List of Threatening Experiences [3]. The list covered nine different adverse events or circumstances: having a major physical or mental health problem; someone close having a major physical or mental health problem; someone close dying; experiencing a natural disaster (e.g., bushfire, flood); job loss, work problems, or study problems; financial problems; relationship problems, including separation or divorce; loneliness or limited social contact; and other (specified). Because we wanted to capture both exposure and perceived impact of each experience, participants responded whether the impact of the event was: a little (1), somewhat (2) or very much (3), with an option of “have not experienced” (0) also provided for each item. We examined the impacts of reporting an adverse experience (any non-zero responses) and the total impact of combined adverse experiences (total score summed across the index). The items were chosen based on adverse experiences that are most likely to influence mental health [3].

We adjusted analyses for self-reported gender (Male/Female/Non-binary), age (in years), education (measured in 10 common education attainment categories converted into years of education), language (English only vs other), history of suspected COVID-19 infection, count of lifetime physical illness diagnoses (from a list of 20 illnesses: hypertension, heart disease, Type 1/2 diabetes, COPD, asthma, emphysema, kidney disease, epilepsy, stroke, multiple sclerosis, Parkinson’s disease, dementia, liver disease, gastrointestinal condition, chronic pain, chronic fatigue syndrome, cancer, life-threatening allergy) and history of a mental health diagnosis by a medical professional (including anxiety, depression, bipolar disorder, schizophrenia, post-traumatic stress disorder, autism spectrum disorder, alcohol or substance use disorder, eating disorder, other).

### Analyses

Linear regression models were first estimated for the main effects of each adverse experience (based on presence or absence) on depression symptoms and anxiety symptoms. Both models included all nine adverse experience items and were adjusted for gender, age, lifetime self-reported mental health diagnosis, and years of education. Two-way interaction terms between the nine adverse experiences (36 total two-way interactions) were then tested for both depression and anxiety models, with only significant interaction terms (*p* < 0.05) retained in the model. The significant interaction terms were determined using stepwise selection (*p* < 0.025 for entry in the model, *p* > 0.05 for removal). Because the presence of adverse experiences was reported on a four-point scale reflecting the impact of the experience (not experienced, little, somewhat, very much), a sensitivity analysis was conducted using an alternative cut-off dichotomizing *not experience* and *little* vs reporting *somewhat* and *very much* impact. In addition, separate linear regression models were run to teste the perceived total impact of adverse experiences (total sum score of the nine items) on depression and anxiety symptoms. All participants (*n* = 1090) who completed the survey were included in the current analyses.

## Results

Table 1 summarises the sample characteristics. The most common adverse experiences reported were financial problems, loneliness, and major physical or mental health problems. The sample’s depression and anxiety scores were elevated overall but typically below clinical cut-offs, with women scoring significantly higher than men on both the PHQ-9 (women: *M* = 8.62, *SD* = 7.23; men: *M* = 5.61, *SD* = 6.68, *t*_*1083*_ = 7.10, *p* < 0.001) and the GAD-7 (women: *M* = 6.67, *SD* = 6.36; men: *M* = 4.35, *SD* = 5.74, *t*_*1083*_ = 6.28, *p* < 0.001). There were 223 women (39%) and 119 men (23%) who scored above the clinical cut-point for depression, and 166 women (29%) and 98 men (19%) who scored above the clinical cut-point for anxiety.

**Table 1:** Sample characteristics

	n	%
Gender		
Male	513	47.1
Female	572	52.5
Non-binary	5	0.5
Mental health diagnosis		
Present	371	34.0
Absent	719	66.0
History of suspected COVID-19 infection		
Present	195	17.9
Absent	895	82.1
Language spoken at home		
English only	923	84.7
Other	167	15.3
Presence of adverse experiences		
Financial problems	697	63.9
Loneliness or limited social contact	685	62.8
Major physical or mental health problem: self	554	50.8
Major physical or mental health problem: close contact	501	46.0
Job loss, work problems, or study problems	427	39.2
Someone close to you dying	397	36.4
Experiencing a natural disaster (e.g., bushfire, flood)	330	30.3
Relationship problems, including separation or divorce	322	29.5
Other adversity	166	15.2

	M	SD
Age	46.96	18.09
PHQ-9 depression score	7.21	7.13
GAD-7 anxiety score	5.59	6.19
Total adversity impact score	6.96	5.95
Count of physical illness diagnoses	2.12	3.26
Years of education	14.29	1.94

Supplementary Table 1 presents the unadjusted relationships between each adverse experience and symptoms of depression and anxiety. As noted, all comparisons were significant, with the presence of each adverse experience associated with a significantly greater level of symptoms (*p* < 0.001).

Table 2 displays the outcomes of the regression analyses testing the independent main effects for each recent adverse experience on depression and anxiety symptoms. Results were similar for both depression and anxiety, reflecting the high correlation between the two outcomes (*r* = 0.89). Financial problems, loneliness, physical or mental health problem (self or close contact) and relationship problems had significant independent main effects on both depression and anxiety symptoms. However, natural disaster, job or study problems and other forms of adversity had no significant independent main effects on symptoms, after accounting for other adverse experiences and potential confounds, while bereavement was only significantly associated with depression symptoms. Among the potential confounding variables included in the models, female gender, mental health diagnosis, greater number of illnesses and younger age were associated with higher symptoms of both depression and anxiety. Sensitivity analyses using an alternative threshold for adverse experiences (“somewhat” or “very much” impact) did not change the outcome of the models presented in Table 2.

**Table 2:** Linear regression models of main effects of adverse experiences on depression and anxiety symptoms

	Depression (PHQ-9)				Anxiety (GAD-7)			
	*b*	SE	t	*p*	*b*	SE	t	*p*
Intercept	3.452	1.475	2.340	0.019	1.697	1.317	1.288	0.198
Recent adverse experiences								
Financial problems	1.013	0.387	2.620	**0.009**	0.970	0.345	2.807	**0.005**
Loneliness or limited social contact	1.915	0.384	4.981	**< 0.001**	1.502	0.343	4.375	**< 0.001**
Major physical or mental health problem: self	3.565	0.422	8.449	**0.001**	2.928	0.377	7.769	**0.000**
Major physical or mental health problem: close contact	0.904	0.395	2.290	**0.022**	0.844	0.353	2.392	**0.017**
Job loss, work problems, or study problems	0.132	0.412	0.320	0.749	0.174	0.368	0.473	0.636
Someone close to you dying	0.774	0.369	2.100	**0.036**	0.583	0.329	1.771	0.077
Experiencing a natural disaster (e.g., bushfire, flood)	− 0.156	0.408	− 0.382	0.702	− 0.519	0.365	− 1.424	0.155
Relationship problems, including separation or divorce	1.772	0.424	4.182	**< 0.001**	1.209	0.379	3.193	**0.001**
Other adversity	0.112	0.509	0.221	0.825	0.291	0.455	0.640	0.522
Gender (female vs male)	1.156	0.339	3.412	**0.001**	0.633	0.303	2.091	**0.037**
Gender (non-binary vs male)	1.386	2.330	0.595	0.552	2.388	2.081	1.148	0.251
Mental health diagnosis	2.309	0.383	6.023	**< 0.001**	2.283	0.342	6.668	**< 0.001**
Count of physical illness diagnoses	0.380	0.054	7.038	**< 0.001**	0.294	0.048	6.086	** < 0.001**
History of suspected COVID-19 infection	0.201	0.422	0.476	0.634	0.002	0.376	0.005	0.996
Age	− 0.041	0.011	− 3.756	**< 0.001**	− 0.044	0.010	− 4.569	**< 0.001**
Years of education	− 0.115	0.084	− 1.382	0.167	0.014	0.075	0.193	0.847
Language (English only vs other)	0.192	0.452	0.425	0.671	0.261	0.404	0.646	0.518

*PHQ-9* Patient Health Questionnaire-9, *GAD-7* Generalized Anxiety Disorder-7

Bold values indicate *p* < .05. b is the unstandardized regression coefficient

The 36 possible two-way interaction terms were tested for addition into the main effects models, using forward stepwise variable selection. For the depression model, none of the interaction terms met inclusion criteria (*p* < 0.025). However, two of the 36 terms were selected based on having significant independent associations with generalized anxiety symptoms as shown in Table 3. Specifically, experiencing both work/study problems and loneliness was associated with significantly higher anxiety (i.e., a synergistic effect). However, experiencing both a natural disaster and relationship problems was associated with less severe anxiety symptoms in the final model (i.e., a buffering effect). In the sensitivity analysis that examined the effect of a higher threshold for adverse experiences (“somewhat” or “very much” impact), the original interactions were no longer significant. However, there were three significant interactions, all indicating buffering effects rather than synergies: between natural disaster and “other” adverse experiences on less severe depression symptoms (*b* = -2.85, *t* = − 3.23, *df* = 1, *p* = 0.001), between health problem and bereavement on less severe depression symptoms (*b* = − 1.81, *t* = − 2.36, *df* = 1, *p* = 0.018) and between loneliness and “other” adverse experiences on less severe anxiety symptoms (*b* = -3.11, *t* = − 3.15, *df* = 1, *p* = 0.002).

**Table 3:** Linear regression model of generalised anxiety symptoms with inclusion of significant interaction terms

	*b*	SE	T	*p*
Intercept	1.601	1.312	1.220	0.223
Adverse experiences				
Financial problems	1.032	0.346	2.982	**0.003**
Loneliness or limited social contact	0.882	0.392	2.252	**0.025**
Major physical or mental health problem: self	2.931	0.375	7.817	**0.000**
Major physical or mental health problem: close contact	0.884	0.351	2.517	**0.012**
Job loss, work problems, or study problems	− 1.261	0.616	− 2.047	**0.041**
Someone close to you dying	0.649	0.329	1.975	**0.048**
Experiencing a natural disaster (e.g., bushfire, flood)	0.146	0.445	0.329	0.742
Relationship problems, including separation or divorce	1.870	0.466	4.012	**< 0.001**
Other adversity	0.384	0.459	0.836	0.404
Interaction effects				
Work/study problems × loneliness	2.012	0.688	2.923	**0.004**
Natural disaster × relationship problems	− 1.830	0.699	− 2.617	**0.009**
Gender (female vs male)	0.566	0.302	1.873	0.061
Gender (non-binary vs male)	2.563	2.070	1.238	0.216
Mental health diagnosis	2.278	0.342	6.668	**< 0.001**
Count of physical illness diagnoses	0.295	0.048	6.126	**< 0.001**
History of suspected COVID-19 infection	0.001	0.375	0.002	0.998
Age	− 0.042	0.010	− 4.405	**< 0.001**
Years of education	0.027	0.074	0.357	0.721
Language (English only vs other)	0.229	0.401	0.569	0.569

Bold values indicate *p* < .05. b is the unstandardized regression coefficient

Finally, the impact of total recent adverse experiences was examined in separate linear regression models, presented in Table 4. The perceived impact of all adverse experiences was strongly associated with depression and anxiety symptoms. Specifically, one standard deviation increase in the perceived impact of combined adversities was associated with an increase of 0.49 standard deviations in depression symptoms (i.e., 3.52 points on the PHQ-9) and 0.46 standard deviations in anxiety symptoms (i.e., 2.87 points on the GAD-7).

**Table 4:** Linear regression models of the perceived impact of combined adversities on depression and anxiety symptoms

	Depression (PHQ-9)				Anxiety (GAD-7)			
	*b*	SE	t	*p*	*b*	SE	t	*p*
Intercept	4.252	1.429	2.976	0.003	2.145	1.273	1.685	0.092
Total adversity score	0.592	0.031	19.191	**< 0.001**	0.482	0.027	17.551	**< 0.001**
Gender (female vs male)	1.261	0.327	3.851	**< 0.001**	0.783	0.292	2.684	**0.007**
Gender (non-binary vs male)	1.063	2.302	0.462	0.644	2.122	2.051	1.035	0.301
Mental health diagnosis	2.600	0.368	7.073	**< 0.001**	2.517	0.327	7.687	**< 0.001**
Count of physical illness diagnoses	0.310	0.053	5.895	**< 0.001**	0.230	0.047	4.919	**< 0.001**
History of suspected COVID-19 infection	0.438	0.416	1.053	0.293	0.197	0.370	0.532	0.595
Age	− 0.045	0.010	− 4.394	**< 0.001**	− 0.045	0.009	− 4.975	**< 0.001**
Years of education	− 0.113	0.082	− 1.377	0.169	0.019	0.073	0.264	0.792
Language (English only vs other)	− 0.163	0.444	− 0.368	0.713	− 0.043	0.396	− 0.110	0.913

*PHQ-9* Patient Health Questionnaire-9, *GAD-7* Generalized Anxiety Disorder-7

Bold values indicate *p* < .05. b is the unstandardized regression coefficient

## Discussion

The present study examined the influence of adverse experiences on depression and anxiety in a representative sample of Australian adults. The first hypothesis was partially supported as seven of the nine adverse experiences had significant, independent, and positive associations with both depression and anxiety. In addition, the perceived impact of the combination of adverse experiences also had a strong association with both depression and anxiety symptoms. The second hypothesis was largely unsupported as only one combination of adverse experiences (work/study problems and loneliness) had a significant association with increased anxiety symptoms. Sensitivity analysis did not alter the finding that synergistic effects of multiple adversities were minimal or absent, with three buffering effects and no synergistic effects observed.

The findings provided little to no support for syndemic theory [22, 27]. None of the adverse biological or social states interacted in their relationships with depression. Only one out of 36 interaction terms (3%) was significantly associated with greater severity of anxiety symptoms, less than what might be expected by chance. However, there were several interactions between adversities that buffered symptoms, in contrast to the predictions of syndemic theory. The overall perceived impact of combined adverse experiences was strongly associated with depression and anxiety symptoms. However, this relationship does not provide a direct test of syndemic theory as it does not examine the intersections between different social or biological conditions on health [21, 29]. In summary, the preponderance of evidence from this study suggests that biological and social adversities did not clearly interact to influence mental health, despite the study accounting for recent experiences in the context of a pandemic and high exposure to natural disasters.

There were some potential challenges in capturing syndemic relationships in the current study. For example, non-linear relationships between different adverse experiences may be challenging to identify. In addition, the varying impact of what appear to be identical adverse experiences, such as bereavement or exposure to natural disaster, may be interpreted and perceived by individuals in vastly subjective ways. Thus, examining such events from a population or ecological perspective loses the subjective detail of an individual’s experience. However, examining from an individual level also comes with challenges in capturing the complexity of discrete events that may differ in both phenomenology and severity, and in accounting for the potential interactions between multiple adverse circumstances. This issue reflects wider difficulties in testing syndemic theory, with major challenges in assessing health outcomes and objectively quantifying social impacts at a population level. As a consequence, syndemics has made limited progress in conclusively demonstrating synergistic disease interactions beyond the original focus on HIV syndemics [27].

Instead of framing mental health outcomes around a syndemics model, a more useful public health perspective might be to treat adverse life events as critical factors, but largely independent experiences that can play a key role in the emergence of symptoms of mental ill-health. That is, although they may co-occur, experiencing *any* single highly adverse social or biological experience may increase vulnerability for experiencing depression or anxiety [5, 13, 26]. The finding that reliable synergies between multiple adverse experiences were not observed in this study is consistent with the broader literature on stressful life events, which indicates that multiple events may or may not have more impact on health outcomes than individual events [5]. Consequently, public health efforts to improve population mental health might be better served by developing tailored responses to discrete adverse experiences, rather than by attempting to develop complex solutions to address theoretically complex circumstances. These might include providing prevention or treatment programs to individuals who experience specific events such as relationship breakdown, unemployment or financial hardship, natural disaster, or bereavement. Appropriate clinical and psychosocial responses could be tailored to the needs of the target group, such as providing housing, employment, and financial supports to those experiencing unemployment or financial distress, or targeted interpersonal therapy for people going through a relationship separation. Further research on intervention targets may benefit from exploring dependencies between unexpected population-level events and their sequelae, such as between natural disasters and loss, or by examining moderators of stress responses to diverse adverse events [12, 13].

### Strengths and limitations

There are several strengths of this study, including its focus on recent adverse experiences (rather than lifetime), measurement and analyses of interactions between multiple social and biological experiences, and the large national sample that was representative based on age, gender and location. However, there are also several limitations of the findings to consider. The study used retrospective reports of adverse experiences and related them to current depression and anxiety symptom levels. Consequently, memory or reporting biases may have influenced self-report of the impacts of events, observed relationships may not be causal, and synergistic effects may unfold over time. The assessment of both perceived impact and exposure to each event may also have influenced how participants responded to the measure. While the focus on recent events may have reduced reporting biases and ensured that multiple adverse events were experienced with temporal proximity, it is possible that longer-term effects of lifetime adversities may lead to different outcomes. Further research on the influence of adverse experiences on mental health may benefit from exploring the timeframe of impacts and whether distal adverse events are more likely to show synergistic relationships. The study focused on both individual- and population-level adverse experiences, with outcomes tested at the individual level. In contrast, syndemic theory is proposed to explain population-level relationships between health and social states. Very few previous studies have been adequately designed to test multilevel interactions, with limited progress made in comprehensively testing the theory. Nevertheless, further research examining community-level health outcomes may provide additional insights into how population adversities and physical health interact with the mental health of the population.

It remains possible that multiple adversities have synergistic impacts on mental health for some people, while for others, contextual factors such as social support may buffer the potential negative impacts of adversity. We did not adjust the models for specific social or health factors, as these may be closely associated with recent experiences of adversity, although such adjustment would have been unlikely to result in greater evidence of synergism. Findings may also have varied if higher thresholds were used to characterise adverse experiences, such as limiting natural disasters to those who lost property or limiting financial problems to those in bankruptcy or unstable housing. Although the sample was recruited to be representative of the Australian adult population on key demographics, it remains possible that there was some self-selection into the study on the basis of elevated symptoms of depression and anxiety. Finally, we did not adjust for multiple comparisons when examining the many interaction terms, although adjusting for Type I error rate would not have changed the conclusion that few interactions were significantly associated with depression or anxiety.

In conclusion, stressful social experiences are strongly associated with increased depression and anxiety symptoms. However, no consistent evidence for synergistic effects of adverse social conditions were observed. Further investigation of the groups most affected by specific adverse experiences may provide greater targeting of public health and clinical supports for people experiencing adversity.

## Supplementary Information

Below is the link to the electronic supplementary material.

Supplementary file1 (DOCX 31 KB)

## Data Availability

Data are available from AD upon request.

## References

[1] Arena AF, Mobbs S, Sanatkar S, Williams D, Collins D, Harris M, Harvey SB, Deady M (2023) Mental health and unemployment: a systematic review and meta-analysis of interventions to improve depression and anxiety outcomes. J Affect Disord 335:450–472 37201898 10.1016/j.jad.2023.05.027

[2] Beaglehole B, Mulder RT, Frampton CM, Boden JM, Newton-Howes G, Bell CJ (2018) Psychological distress and psychiatric disorder after natural disasters: systematic review and meta-analysis. Br J Psychiatry 213:716–722 30301477 10.1192/bjp.2018.210

[3] Brugha TS, Cragg D (1990) The list of threatening experiences: the reliability and validity of a brief life events questionnaire. Acta Psychiatr Scand 82:77–81 2399824 10.1111/j.1600-0447.1990.tb01360.x

[4] Butterworth P, Cherbuin N, Sachdev P, Anstey KJ (2012) The association between financial hardship and amygdala and hippocampal volumes: results from the PATH through life project. Soc Cogn Affect Neurosci 7:548–556 21551226 10.1093/scan/nsr027 PMC3375885

[5] Cohen S, Murphy MLM, Prather AA (2019) Ten surprising facts about stressful life events and disease risk. Annu Rev Psychol 70:577–597 29949726 10.1146/annurev-psych-010418-102857 PMC6996482

[6] Covid-Mental Disorders Collaborators (2021) Global prevalence and burden of depressive and anxiety disorders in 204 countries and territories in 2020 due to the COVID-19 pandemic. Lancet 398:1700–1712 34634250 10.1016/S0140-6736(21)02143-7 PMC8500697

[7] Delport A, Tabana H, Knight L, Wouters E (2022) The co-occurrence of the SAVA syndemic, depression and anxiety as barriers to antiretroviral therapy adherence among sub-Saharan Africa population groups: a scoping review protocol. PLoS ONE 17:e0274614 36126067 10.1371/journal.pone.0274614 PMC9488813

[8] Faravelli C, Lo Sauro C, Lelli L, Pietrini F, Lazzeretti L, Godini L, Benni L, Fioravanti G, Talamba GA, Castellini G, Ricca V (2012) The role of life events and HPA axis in anxiety disorders: a review. Curr Pharm Des 18:5663–5674 22632471 10.2174/138161212803530907

[9] Fronteira I, Sidat M, Magalhaes JP, de Barros FPC, Delgado AP, Correia T, Daniel-Ribeiro CT, Ferrinho P (2021) The SARS-CoV-2 pandemic: a syndemic perspective. One Health 12:100228 33614885 10.1016/j.onehlt.2021.100228 PMC7887445

[10] Guan N, Guariglia A, Moore P, Xu F, Al-Janabi H (2022) Financial stress and depression in adults: a systematic review. PLoS ONE 17:e0264041 35192652 10.1371/journal.pone.0264041 PMC8863240

[11] Illangasekare S, Burke J, Chander G, Gielen A (2013) The syndemic effects of intimate partner violence, HIV/AIDS, and substance abuse on depression among low-income urban women. J Urban Health 90:934–947 23529665 10.1007/s11524-013-9797-8 PMC3795184

[12] Kendler KS, Karkowski LM, Prescott CA (1999) Causal relationship between stressful life events and the onset of major depression. Am J Psychiatry 156:837–841 10360120 10.1176/ajp.156.6.837

[13] Kessler RC (1997) The effects of stressful life events on depression. Annu Rev Psychol 48:191–214 9046559 10.1146/annurev.psych.48.1.191

[14] Kneale D, Bécares L (2023) The influence of a hostile environment on a syndemic of depression, stress and chronic limiting illness among LGBTQ+ people during the COVID-19 pandemic. Sociol Health Illness 46:114–136 10.1111/1467-9566.13689 37395723

[15] Kroenke K, Spitzer RL, Williams JB, Lowe B (2010) The Patient Health Questionnaire Somatic, Anxiety, and Depressive Symptom Scales: a systematic review. Gen Hosp Psychiatry 32:345–359 20633738 10.1016/j.genhosppsych.2010.03.006

[16] Leach LS, Butterworth P, Olesen SC, Mackinnon A (2013) Relationship quality and levels of depression and anxiety in a large population-based survey. Soc Psychiatry Psychiatr Epidemiol 48:417–425 22875222 10.1007/s00127-012-0559-9

[17] McLaughlin KA, Hatzenbuehler ML (2009) Stressful life events, anxiety sensitivity, and internalizing symptoms in adolescents. J Abnorm Psychol 118:659–669 19685962 10.1037/a0016499 PMC2881589

[18] Mendenhall E, Kohrt BA, Norris SA, Ndetei D, Prabhakaran D (2017) Non-communicable disease syndemics: poverty, depression, and diabetes among low-income populations. Lancet 389:951–963 28271846 10.1016/S0140-6736(17)30402-6 PMC5491333

[19] Rehm J, Shield KD (2019) Global burden of disease and the impact of mental and addictive disorders. Curr Psychiatry Rep 21:10 30729322 10.1007/s11920-019-0997-0

[20] Shim RS, Starks SM (2021) COVID-19, structural racism, and mental health inequities: policy implications for an emerging syndemic. Psychiatr Serv 72:1193–1198 33622042 10.1176/appi.ps.202000725

[21] Singer M, Bulled N, Ostrach B, Mendenhall E (2017) Syndemics and the biosocial conception of health. Lancet 389:941–950 28271845 10.1016/S0140-6736(17)30003-X

[22] Singer M, Clair S (2003) Syndemics and public health: reconceptualizing disease in bio-social context. Med Anthropol Q 17:423–441 14716917 10.1525/maq.2003.17.4.423

[23] Spitzer RL, Kroenke K, Williams JB (1999) Validation and utility of a self-report version of PRIME-MD: the PHQ primary care study. Primary Care Evaluation of Mental Disorders. Patient Health Questionnaire. JAMA 282:1737–1744 10568646 10.1001/jama.282.18.1737

[24] Spitzer RL, Kroenke K, Williams JB, Lowe B (2006) A brief measure for assessing generalized anxiety disorder: the GAD-7. Arch Intern Med 166:1092–1097 16717171 10.1001/archinte.166.10.1092

[25] Sweetland AC, Kritski A, Oquendo MA, Sublette ME, Norcini Pala A, Silva LRB, Karpati A, Silva EC, Moraes MO, Silva J, Wainberg ML (2017) Addressing the tuberculosis-depression syndemic to end the tuberculosis epidemic. Int J Tuberc Lung Dis 21:852–861 28786792 10.5588/ijtld.16.0584 PMC5759333

[26] Tennant C (2002) Life events, stress and depression: a review of recent findings. Aust N Z J Psychiatry 36:173–182 11982537 10.1046/j.1440-1614.2002.01007.x

[27] Tsai AC, Mendenhall E, Trostle JA, Kawachi I (2017) Co-occurring epidemics, syndemics, and population health. Lancet 389:978–982 28271848 10.1016/S0140-6736(17)30403-8 PMC5972361

[28] Uher R, Zwicker A (2017) Etiology in psychiatry: embracing the reality of poly-gene-environmental causation of mental illness. World Psychiatry 16:121–129 28498595 10.1002/wps.20436 PMC5428165

[29] Weiss NM (2021) A critical perspective on syndemic theory and social justice. Public Anthropologist 3:285–317

[30] Zisook S, Iglewicz A, Avanzino J, Maglione J, Glorioso D, Zetumer S, Seay K, Vahia I, Young I, Lebowitz B, Pies R, Reynolds C, Simon N, Shear MK (2014) Bereavement: course, consequences, and care. Curr Psychiatry Rep 16:482 25135781 10.1007/s11920-014-0482-8

