# Supplementary material for: Testing a syndemics perspective on the effects of multiple adversities on depression and anxiety symptoms in a representative population sample
Source: Soc Psychiatry Psychiatr Epidemiol. 2024 Mar 14;59(11):2009–17. doi: 10.1007/s00127-024-02638-w (PMC11522096; doi:10.1007/s00127-024-02638-w)
Supplement: Supplementary file 1 — Supplementary file1 (DOCX 31 KB) [file 127_2024_2638_MOESM1_ESM.docx]

Supplementary Table 1: Prevalence of adverse experiences and unadjusted relationships with depression and anxiety symptoms

|  | Experienced adversity | | | | |  | Did not experience adversity | | | | |
| --- | --- | --- | --- | --- | --- | --- | --- | --- | --- | --- | --- |
|  | n | PHQ-9 mean (SD) | | GAD-7 mean (SD) | |  | n | PHQ-9 mean (SD) | | GAD-7 mean (SD) | |
| Financial problems | 849 | 9.08 | ( 7.16 ) | 7.18 | ( 6.35 ) |  | 557 | 3.72 | ( 5.42 ) | 2.58 | ( 4.37 ) |
| Loneliness or limited social contact | 859 | 9.36 | ( 7.10 ) | 7.39 | ( 6.24 ) |  | 547 | 3.19 | ( 4.97 ) | 2.19 | ( 4.17 ) |
| Major physical or mental health problem: self | 688 | 10.87 | ( 7.11 ) | 8.67 | ( 6.28 ) |  | 718 | 3.22 | ( 4.44 ) | 2.20 | ( 3.75 ) |
| Major physical or mental health problem: close contact | 618 | 10.38 | ( 7.16 ) | 8.27 | ( 6.23 ) |  | 789 | 4.28 | ( 5.63 ) | 3.08 | ( 4.87 ) |
| Job loss, work problems, or study problems | 524 | 10.32 | ( 7.07 ) | 8.32 | ( 6.26 ) |  | 882 | 4.96 | ( 6.20 ) | 3.60 | ( 5.23 ) |
| Someone close to you dying | 483 | 9.82 | ( 7.44 ) | 7.82 | ( 6.43 ) |  | 921 | 5.47 | ( 6.32 ) | 4.08 | ( 5.47 ) |
| Experiencing a natural disaster (e.g., bushfire, flood) | 397 | 10.41 | ( 7.04 ) | 8.24 | ( 6.20 ) |  | 1010 | 5.60 | ( 6.55 ) | 4.23 | ( 5.64 ) |
| Relationship problems, including separation or divorce | 400 | 11.34 | ( 7.23 ) | 9.00 | ( 6.35 ) |  | 1004 | 5.21 | ( 6.14 ) | 3.91 | ( 5.32 ) |
| Other adversity | 164 | 11.48 | ( 7.17 ) | 9.16 | ( 6.05 ) |  | 924 | 6.43 | ( 6.86 ) | 4.95 | ( 6.00 ) |

*Notes*: PHQ-9: Patient Health Questionnaire-9; GAD-7: Generalized Anxiety Disorder-7; *p*<.001 for all unadjusted comparisons
